# Genetics in psychiatry: common variant association studies

**DOI:** 10.1186/2040-2392-1-6

**Published:** 2010-03-25

**Authors:** Joseph D Buxbaum, Simon Baron-Cohen, Bernie Devlin

**Affiliations:** 1 Seaver Autism Center for Research and Treatment and Department of Psychiatry, Mount Sinai School of Medicine, New York, NY, USA; 2 Autism Research Centre, Department of Psychiatry, Cambridge University, Cambridge, UK; 3 Department of Psychiatry, University of Pittsburgh School of Medicine, Pittsburgh, Pennsylvania, USA

## Abstract

Many psychiatric conditions and traits are associated with significant heritability. Genetic risk for psychiatric conditions encompass rare variants, identified due to major effect, as well as common variants, the latter analyzed by association analyses. We review guidelines for common variant association analyses, undertaking after assessing evidence of heritability. We highlight the importance of: suitably large sample sizes; an experimental design that controls for ancestry; careful data cleaning; correction for multiple testing; small P values for positive findings; assessment of effect size for positive findings; and, inclusion of an independent replication sample. We also note the importance of a critical discussion of any prior findings, biological follow-up where possible, and a means of accessing the raw data.

## Introduction

Extensive evidence suggests that many psychiatric conditions have a substantial genetic component. A long-standing perspective in the field of psychiatric genetics is that a significant proportion of risk arises from common variants, including single nucleotide polymorphisms (SNPs) (see a general review of common variants in Iyengar and Elston, 2007 [1]). Genome-wide and candidate-gene association studies represent means of testing for common variants contributing to risk. Genome-wide studies take an unbiased (i.e., non-hypothesis-driven) view of the genome but bring with them a heavy burden in terms of correction for multiple testing. In contrast, in the typical candidate-gene association study, genes are selected based on their function and/or position (if there is positional information from linkage, chromosomal rearrangements or copy number variant studies) and as such are hypothesis-driven. In both cases, the design, execution, analysis and interpretation of the studies are crucial. The current commentary will address some of the relevant issues.

Ten years ago, *Nature Genetics*, recognizing the difficulties in replicating candidate gene association findings in complex conditions or phenotypes, published an editorial [2] that proposed criteria for acceptance of such studies. Their recommendations were summarized as follows:

"Ideally, they should have large sample sizes, small P values, report associations that make biological sense and alleles that affect the gene product in a physiologically meaningful way. In addition, they should contain an initial study as well as an independent replication, the association should be observed both in family based and population-based studies, and the odds ratio and/or attributable risk should be high" (pp. 1-2).

While these remain excellent guidelines, advances over the course of the past decade are such that these guidelines need to be revisited. First and foremost, genotyping has become much more efficient, widespread, and less expensive. For this reason genome-wide association studies are more common. On the other hand, most laboratories with access to clinical samples can now carry out smaller (candidate gene) association studies. Second, empirical data have dramatically re-enforced the fundamental concerns that precipitated the *Nature Genetics *editorial, i.e., that genetic association studies have mostly (but not entirely) failed to replicate. Third, for many conditions, samples are available through international repositories. And, fourth, we have greater understanding of sources of error in association studies, as well as how to best address them.

The widespread availability of less expensive genotyping and the access to international DNA repositories do not need further elaboration here, and in relation to autism we would direct the reader to the NIMH http://www.nimhgenetics.org/, AGRE http://www.agre.org and Simons Foundation http://sfari.org/ websites. The empirical data showing just how extensive the problems are warrant brief review, as do some of the evolving methods to address them.

## A core concern

In 2002, Hirschhorn et al. [3] surveyed 600 positive association studies in medical genetics broadly (i.e., not just autism) and focused on 166 genes that had been studied three or more times. One distressing conclusion was that only 6 of these 166 were consistently replicated (defined as a positive association in at least 75% of the follow-up studies). The news was not all bad, because well over half of the remaining initial, positive associations were replicated at least once. Some of the most consistent findings include *APOE *in Alzheimer disease, *INS *in type I diabetes, *CCR5 *in HIV infection, *PRNP *in Creutfeldt-Jakob disease, *CTLA4 *in Graves' disease, and *F5 *in deep vein thrombosis.

In a follow-up analysis, 301 studies covering 25 different associations were studied in greater detail [4]. Pooled analysis of the follow-up studies supported evidence for association by meta-analysis in 8 out of the 25 associations indicating that there were very modest, but likely real, effects in about a third of the initial, positive reports. Two of these positive replications were in psychiatric conditions (schizophrenia in both, a disease with an estimated heritability of 70-85% [5]), and the estimated effect sizes should be noted: 1.12 for *DRD3 *(with a 95% confidence interval of 1.02-1.23 with a fixed effect model) and 1.07 for *HTR2A *(95% confidence interval of 1.01-1.14). Most recent analyses of these (and other) candidate genes in schizophrenia by meta-analysis find less support, if any, for association [6,7]. In any case, for genes positive by meta-analysis, odds ratios are all almost universally below 1.5 and typically below 1.25.

These effect sizes are consistent with both those derived from studies in model organisms (usually mice) and those now arising from genome-wide studies (per-allele odds ratios less than 1.5, http://www.genome.gov/gwastudies/) [8]. Going into a genetic study with a realistic expectation of likely effect size will go a long way towards addressing limitations in many association analyses. Association studies in autism (with estimates of heritability above 90%, [5]) are not immune to the problems with candidate gene association studies. Out of more than 100 published association studies in autism and associated conditions, less than 10 have been consistently replicated (see http://wren.bcf.ku.edu/ and http://gene.sfari.org/).

## Updating the recommendations

Given this failure to replicate, we feel it would be useful to elaborate on the recommendations made a decade ago, as follows:

*"large sample sizes" *- In light of the modest effect sizes anticipated in any study in psychiatric genetics, power becomes a serious concern. Investigators now recognize that a genome-wide study requires sample sizes on the order of several thousand cases and controls or trios to ensure detection of some common variants of modest effect, unless the circumstances are exceptional. Candidate gene studies are not much different, even if the significance level for P-value used for the test is larger. For example, even for effects on the high side of the allelic odds ratio (i.e., near 1.5), sample sizes of 500 or more cases and controls or trios would be required to have good power to reject the null hypothesis - even if one of the genotyped and tested variants is a risk variant. When the true odds ratio approaches 1.1, the required sample size is roughly an order of magnitude larger. Note, moreover, that these kinds of power calculations ignore the prior probability that any selected gene contains a risk variant. This probability is likely to be small, given the limited scope of the study and the findings regarding replication described earlier. Large sample sizes are warranted even in the case of a replication study where the first study gives a higher estimated effect size, as it is likely that the estimated effect size from the primary study represents an overestimate (sometimes referred to as "winner curse").

*"small P values" *- While small P values remain important (with small implying P much less than 0.05), a focus on P values alone is inadequate. A consideration of effect size is always required (see below), and identical P values in different studies "can have different implications for the plausibility of a true association depending on the factors that affect the power of the test, such as the minor allele frequency (MAF) of the SNP and the size of the study" [9]. An underpowered test can produce a small P value that in fact provides little evidence against the null hypothesis. This gets back to avoiding under-powered tests while scrutinizing the genotype results to remove poorly-performing SNPs and SNPs with a low minor allele frequency.

*Data cleaning needs to be both rigorous and reported*. In addition to removing markers with low minor allele frequency, markers and samples with higher failure rates need to be removed, as do markers that fail Hardy-Weinberg testing or cluster poorly. Data should be analyzed for cryptic duplicates and Mendelian and sex inconsistencies. Finally, some sense of genotype error rates should be presented. Note that there is no "perfect" approach to data cleaning, and it is difficult to determine a priori the impact of poor quality data. It might have little noteworthy effect in a single data set, yet the effect could be compounded when numerous data sets are combined, such as by meta-analysis. Making raw data available for scrutiny and meta-analysis, increasingly the norm in genome-wide studies and in studies making use of public DNA repositories, is therefore encouraged.

*Correction for multiple testing*. While the standard in genome-wide studies, candidate gene studies often fail to provide correction for multiple testing. Reporting a nominal P value that is likely the result of chance is not accepted in science as a whole and should not be a part of psychiatric genetic research. With the advent of DNA repositories it is becoming much harder to provide sample-wide correction, but certainly experiment-wide correction is to be expected. With software programs like PLINK and UNPHASED now available for permutation testing [10,11], running such corrections should become the norm.

*"report associations that make biological sense and alleles that affect the gene product in a physiologically meaningful way" *- This remains an ideal but has a different interpretation in genome-wide studies. In genome-wide studies the hypothesis-independent approach can lead to novel findings that do not make sense in the current conceptualization of a condition or phenotype, even if true. And for psychiatric conditions it is possible to develop an ad hoc story based only on the fact that a gene is expressed in the brain. So, the criterion of biological plausibility is a bonus but not necessarily a requirement. Follow-up analyses (or an *a priori *focus) on functional alleles can help to making biological sense of the association data. That is, if a genetic finding gives rise to a physiologically well-specified hypothesis that can be tested beyond a genetic design, this constitutes stronger converging evidence.

*"they should contain an initial study as well as an independent replication" *- Including replications in the same report is the sine qua non for a meaningful conclusion. With the advent of DNA repositories it has become harder to conceive of a study of a common categorical trait that cannot avail itself to a replication sample. The emphasis here is on the word 'independent', since a within-sample replication may not be sufficient (as phenotyping or genotyping errors may be common to both halves of a split sample).

In addition, if there are other studies of the same locus in the literature, careful analysis of sample overlap needs to be described to ensure the principle of independence. Furthermore, the extent to which the study being presented replicates the prior findings should be discussed, and should address whether the same marker (or a marker in linkage disequilibrium) is showing the same effect in the same direction.

*"the association should be observed both in family based and population-based studies" *- The requirement for a family-based cohort arose from the potential for false-positive findings due to population stratification. With the advent of methods that adjust for ancestry, it is more relevant to expect the design to control for ancestry by either a family-based design or sufficient number of markers to estimate and adjust for ancestry. In general, case-control studies should include matching based on ancestry using standard approaches [12-14]; and with the reduced cost of genotyping, this is now an expectation in all well-designed studies.

*"the odds ratio and/or attributable risk should be high" *- This criterion warrants some elaboration. Empirical data indicate that for many conditions and phenotypes, high odds ratios and high attributable risk is the exception, not the norm. It is for this reason that the field of psychiatric genetics has moved to ever-increasing sample sizes. It is more appropriate to expect that, for a suitably-powered study, some measure of effect size be provided. The replication sample gives an opportunity to estimate effect size, which is likely - due to winner's curse - to be much more accurate than such an estimate from the discovery sample.

## Summary

Many psychiatric conditions are associated with significant heritability [5], and many psychology traits, used as endophenotypes or intermediate phenotypes, have appreciable heritability as well. While there is emerging evidence that a proportion of risk for many psychiatric conditions resides in rare variants of major effect, there is also compelling evidence for common variants, most recently from independently replicated findings from genome-wide association studies in schizophrenia and autism [15-18]. With the advent of large, publically available samples and inexpensive genotyping, the guidelines proposed ten years ago are now a target that is accessible to most laboratories. Advances in the past decade have also led to improved approaches to supplement these guidelines. Taken together, a well-designed and executed association study should have the following components:

• Evidence of heritability for the condition or trait

• Suitably large sample sizes, in the context of expected effect sizes and power

• Standardized, careful data cleaning, described in detail

• Correction for multiple testing

• Inclusion of an independent replication sample

• Experimental design that controls for ancestry by either a family-based design or by the inclusion of a sufficient number of markers to estimate and adjust for ancestry

• Small P values for positive findings

• Presentation of metrics of effect size for positive findings

• A critical discussion of any prior findings

• Biological follow-up (either experimental or, minimally, in discussion)

• A means of accessing the raw data

## Competing interests

JDB and SBC are Editors-in-Chief of *Molecular Autism*.
